# The Complete Chloroplast Genome Sequences of the Medicinal Plant *Pogostemon cablin*

**DOI:** 10.3390/ijms17060820

**Published:** 2016-06-06

**Authors:** Yang He, Hongtao Xiao, Cao Deng, Liang Xiong, Jian Yang, Cheng Peng

**Affiliations:** 1 State Key Laboratory Breeding Base of Systematic Research, Development and Utilization of Chinese Medicine Resources, Chengdu University of Traditional Chinese Medicine, Chengdu 611137, China; hawkheyang@163.com (Y.H.); xiling0505@126.com (L.X.); 2 School of Medicine, University of Electronic Science and Technology of China, Chengdu 610072, China; xht927@163.com; 3 Department of Pharmacy, Hospital of the University of Electronic Science and Technology of China and Sichuan Provincial People’s Hospital, Chengdu 610072, China; 4 Department of Bioinformatics, DNA Stories Bioinformatics Center, Chengdu 611731, China; brentcaodeng@gmail.com (C.D.); yangjian@dnastories.com (J.Y.)

**Keywords:** *Pogostemon cablin*, sequencing, chloroplast genome, SSR, phylogenetic analysis

## Abstract

*Pogostemon cablin*, the natural source of patchouli alcohol, is an important herb in the *Lamiaceae* family. Here, we present the entire chloroplast genome of *P. cablin*. This genome, with 38.24% GC content, is 152,460 bp in length. The genome presents a typical quadripartite structure with two inverted repeats (each 25,417 bp in length), separated by one small and one large single-copy region (17,652 and 83,974 bp in length, respectively). The chloroplast genome encodes 127 genes, of which 107 genes are single-copy, including 79 protein-coding genes, four rRNA genes, and 24 tRNA genes. The genome structure, GC content, and codon usage of this chloroplast genome are similar to those of other species in the family, except that it encodes less protein-coding genes and tRNA genes. Phylogenetic analysis reveals that *P. cablin* diverged from the *Scutellarioideae* clade about 29.45 million years ago (Mya). Furthermore, most of the simple sequence repeats (SSRs) are short polyadenine or polythymine repeats that contribute to high AT content in the chloroplast genome. Complete sequences and annotation of *P. cablin* chloroplast genome will facilitate phylogenic, population and genetic engineering research investigations involving this particular species.

## 1. Introduction

*Lamiaceae*, the mint family of flowering plants, is the largest family of the *Lamiales* order which is composed of more than 7000 species [1]. Plants from this family are valued for its flavor, fragrance, and medicinal properties. *Pogostemon* is a large genus belonging to this family, among which patchouli (*Pogostemon cablin*) is one of the best-known members. *P. cablin* is an annual herb native to the Philippines [2], and has been widely cultivated in tropical and subtropical areas of Asia [3]. Chemical and pharmacological studies of *P. cablin* in the last few decades indicate that patchouli consists of more than 40 major components, including monoterpenoids and sesquiterpenoids [4], triterpenoids and steroids [5], flavonoids [6], and alkaloids and phenylpropanoid glycosides [7]. The abundant patchouli alcohol in its leaves is an important ingredient for perfumes, incense, soaps, and cosmetic products [8,9]. It also exerts a wide range of medicinal effects, including inhibition of platelet aggregation, anti-inflammatory activity, as well as antidepressant, aphrodisiac, febrifuge, astringent, carminative, diuretic, sedative, and tonic properties [10,11]. Furthermore, patchouli is also an essential Chinese medicinal plant [12] that can be used to eliminate heat and dampness, relieve fatigue, and cure indigestion, headache, and fever which are documented in Chinese Pharmacopoeia [13].

Molecular sequences provide vast information not only about genes and its encoded proteins, but also functional implications and the evolutionary relationships. The development of next-generation sequencing technologies has allowed for the sequencing of entire chloroplast genomes. In angiosperm, chloroplast genomes are mostly circular DNA molecules with a characteristic quadripartite structure that is comprised of two inverted repeats (IRs, about 20–28 kb) and two single copy regions (large single-copy region, LSC, 80–90 kb; small single-copy region, SSC, 16–27 kb). The genetic composition in angiosperm chloroplast genomes is relatively conserved which encodes four rRNAs, ~30 tRNAs, and ~80 single-copy proteins [14]. Within the Lamiaceae family, the complete chloroplast genomes of several plants have been published [15,16,17], thereby providing additional evidence for the conservation and evolution of chloroplast genomes. Nevertheless, no chloroplast genome belonging to genus *Pogostemon* has been reported. Few data are available in respect of the *P. cablin* chloroplast genome. In the present study, we report the complete chloroplast genome of *P. cablin* produced by Illumina HiSeq platform (Illumina, San Diego, CA, USA). Further annotation revealed information regarding the conservation and variation of the genome compared with other *Lamiaceae* species. Phylogenetic analysis also establishes the evolutionary position of this particular plant species. These data may lead to a better understanding of evolutionary history of the *Lamiaceae* clade and facilitate phylogenic, population, and genetic engineering research regarding this important medicinal plant.

## 2. Results and Discussions

### 2.1. Genome Features

**Figure 1:**
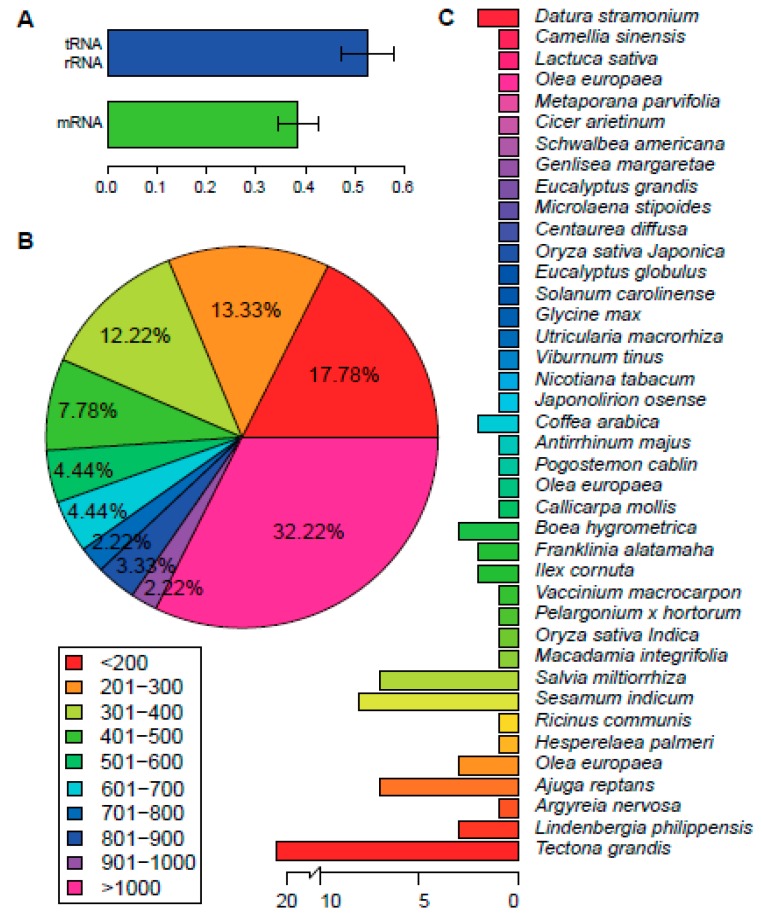
Genome Annotation. (**A**) GC content of protein-coding genes and tRNA/rRNA genes; (**B**) the length distribution of protein-coding genes; (**C**) the number of best hits for *P. cablin* chloroplast protein-coding genes among species.

Illumina Hiseq 2500 platform (Illumina, San Diego, CA, USA) was used to generate 2.44 GB pair end reads (2 × 125 bp). Clean reads were obtained by removing adaptors and low-quality read pairs. In total, we got 9,743,972 clean read pairs. Velvet assembler assembled a total of 40 contigs with N50 length of 25,662 bp (List 1). The contigs were then mapped to the chloroplast genome of *Salvia miltiorrhiza* to verify orders and orientations (Supplementary Table S1). Gap closing procedures was performed by PCR amplification of the gap regions and followed by Sanger sequencing (Supplementary Figure S1; designed primers are shown in Supplementary Table S2). Finally, we obtained the complete chloroplast genome of *P. cablin*, which is comprised of 152,460 bp, that falls within the range of the typical size of an angiosperm chloroplast genome [14]. We verified the genome by comparing this with the chloroplast genome of *Salvia miltiorrhiza*, thereby confirming the absence of disorders or reversion in the genome. The genome exhibited a general quadripartite structure of plants, with two reverse repeated regions (IRa and IRb) of 25,417 bp in length. The repeat regions divided the genome into two single-copy regions, SSC and LSC with 17,652 and 83,974 bp in length, respectively. The GC content of *P. cablin* chloroplast genome is 38.24%, which is in line with that of other asterid chloroplast genomes [15,16,18,19,20]. The GC content of IR regions is 43.5%, which is higher compared with GC content in LSC and SSC regions (36.3% and 32.1%, respectively). The relatively high GC content of IR regions was mostly attributable to the rRNA genes and tRNA genes. These findings were also in agreement those of *Salvia miltiorrhiza* [16]. Intriguingly, the high GC-content regions are primarily found in tRNA genes and rRNA genes, regardless of whether the genes are located in IR regions or not (Figure 1A; Supplementary Table S3), whereas the protein-coding genes mostly possess relatively low GC content.

#### **List 1.** Summary of contigs.

Contig Number40Total size150,312Average length3757.8GC Number57,886AT number92,426N50 length25,662Minimum contig length515Maximum contig length26,687

### 2.2. Genome Annotations

**Figure 2:**
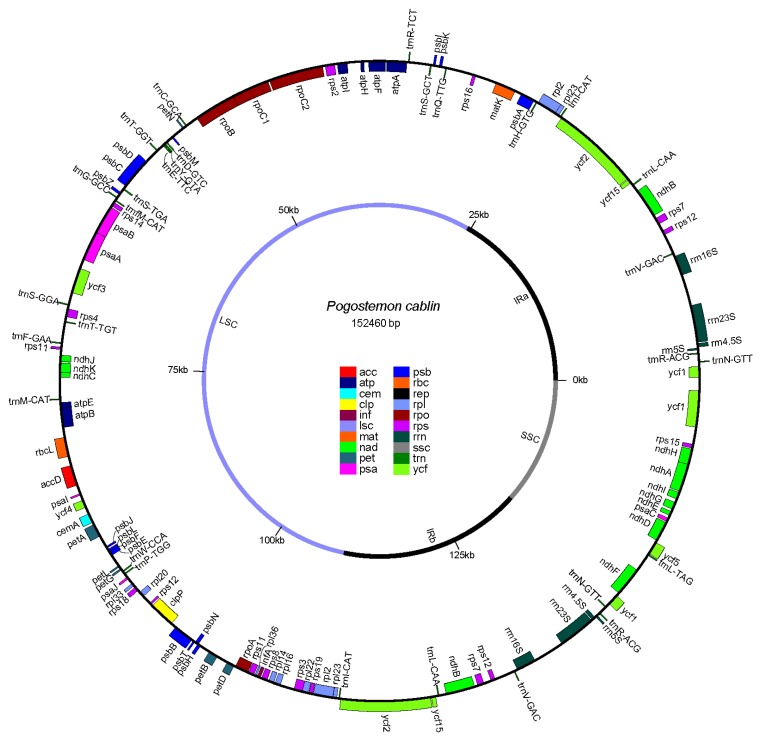
Genome schema of the *P. cablin* chloroplast genome. Genes inside the circle are transcribed clockwise, and counterclockwise transcribed otherwise. Fill colors represent different functional groups that specific genes fall into.

The chloroplast genome of *P. cablin* was predicted to consist of 127 genes, which encodes 107 genes, including four rRNA genes, 24 tRNA genes, and 79 protein-coding genes (Figure 2, Supplementary Table S3). Approximately 57.0%, 6.2% and 1.4% of the genome sequence produce protein, rRNA, and tRNA, respectively. The two IR regions contain five tRNA genes, eight protein-coding genes and all four rRNA genes. The LSC region contains 17 tRNA genes and 63 protein-coding genes, while the SSC region consists of one tRNA gene and 11 protein-coding genes. The components of the IR regions, LSC region, and SSC region are slightly different from that of *Salvia miltiorrhiza* and *Origanum vulgare* L. The chloroplast genome of *P. cablin* has more protein-coding genes and less tRNA genes in LSC regions, while the two IR regions harbor more protein-coding genes, as well as more tRNA genes compared to *Salvia miltiorrhiza* [5] and *Origanum vulgare* L. [17]. There are 11 introns distributed in nine genes that contained one or more introns (Table 1). Two genes (*ycf3* and *clpP*) contain two introns and seven other genes comprise one intron, which was 1055 bp in length and was detected in the *ndhA* gene.

There are 29 unique tRNA genes in chloroplast genome of *P. Cablin*, which transports 19 amino acids for protein biosynthesis. The gene coding trnK-UUU is missing from the current annotation. This gene, which contains a large intron that codes *matK* gene, was detected in the chloroplast genome of *Salvia miltiorrhiza*, *Origanum vulgare* L., and *Premna microphylla*. The *matK* gene is present in chloroplast genome of *P. cablin* in exactly the same order as that in *Salvia miltiorrhiza* and *Origanum vulgare* L. This indicates that the *trnK-UUU* gene should be present in chloroplast genome of *P. cablin*, but not identified, or maybe it diverged earlier from *trnK-UUU* gene in other species. To verify this hypothesis, we also annotated the genome by DOGMA. The results show that a part of the *trnK-UUU* gene is present in chloroplast genome of *P. cablin*, namely, from 29,770 to 29,806. However, it is merely about 40 bp in size and is apparently not the full-length gene.

The 79 unique genes that encode proteins comprise 81,060 bp that encoded for 27,020 codons. We further computed the codon usage frequency of the *P. cablin* chloroplast genome. Of these codons, 2916 (10.8%) encode leucine, whereas just 319 (1.2%) encode cysteine (Table 2). The two are the most and least frequently used amino acids in *P. Cablin* chloroplast genome, respectively. However, we did not find any tRNA gene that transports lysine, and the usage of this amino acid is the same as that in *Salvia miltiorrhiza* (5.3% in *P. cablin* and 5.4% in *Salvia miltiorrhiza*).

**Figure 3:**
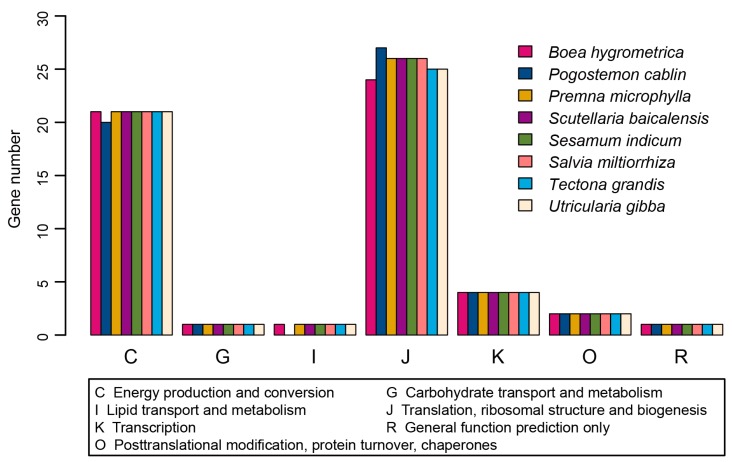
The COG (Clusters of Orthologous Groups) classification and distribution of genes in different species.

There are 90 protein-coding genes, of which nine are duplicated (Supplementary Table S3). The length distribution of protein-coding genes was shown in Figure 1B. Two genes, *rps12* and *ycf1*, occurred as three copies. Both are duplicated in two IR regions, as well as in the LSC or SSC region, respectively. This is the same as that in *Salvia miltiorrhiza* and *Origanum vulgare* L. Meanwhile, the *rps11* gene has two copies in the *P. cablin* chloroplast genome. One copy is located in LSC region, together with a cluster of other genes, whereas the other copy is located in the LSC region, specifically flanking the *trnF-GAA* gene. The location and orientation of the former copy are the same as that in *Salvia miltiorrhiza* and *Origanum vulgare* L., whereas the later copy, which is shorter, seems to be unique to the *P. cablin* chloroplast genome. We then mapped the proteins to the nr, Clusters of Orthologous Groups (COG), and Kyoto Encyclopedia of Genes and Genomes (KEGG) database. Homologs of all the 79 unique genes except for *accD* were identified in the nr database; 67 of these showed homologies in KEGG database; and just 38 of the genes could be assigned to COG (Tables S4–S6). The proteins coded by these genes have their best hit in 40 species in the nr database (Figure 1C). The species with the highest number of best hits was *Tectona grandis*, which belongs to the same family as *P. cablin*. Most of the proteins are involved in photosystems I and II or the ribosomal proteins, as indicated by mapping with nr database. Mapping to KEGG database also confirmed these results. The COG annotation of these proteins mainly fell into two functional classes: energy production and conversion (Category C) or translation, ribosomal structure and biogenesis (Category J) (Figure 3). This is in line with other species in the same order [6,16]. Mapping chloroplast genes from seven other species in *Lamiales* to COG database also revealed the same tendency (Figure 3; Supplementary Table S7).

### 2.3. Phylogenetic Analysis and Divergence Time Estimation

**Figure 4:**
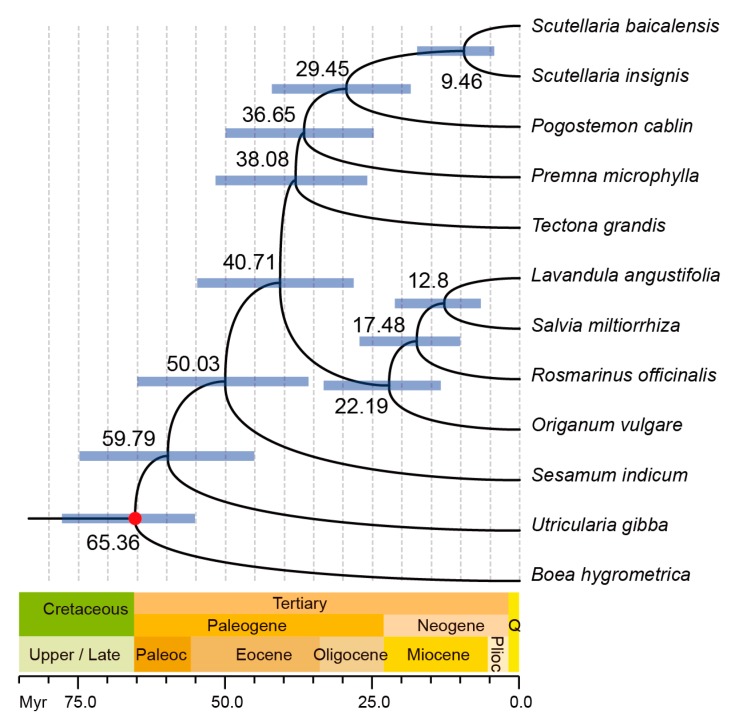
Phylogenetic tree reconstructed based on chloroplast genome alignments from several species. The purple bars at the nodes indicate 95% posterior probability intervals, while the red dot correspond to the calibration point.

To identify the evolutionary position of *P. cablin* within *Lamiaceae*, we carried out multiple sequence alignments using the whole chloroplast genome sequences of eight other sequenced chloroplast genomes in *Lamiaceae*. Three species from different family in *Lamiales* were also chosen as outgroup. Figure 4 shows that the four *Nepetoideae* plants compose a unique clade, whereas the other five plants are relatively divergent. *P. cablin* is close to *Premna microphylla* and the clade of *Scutellarioideae* comprised *Scutellaria insignis* and *Scutellaria baicalensis*. All the times on nodes matched well with the data deposited in TIMETREE [21], a public knowledge-base of divergence times among organisms, thereby confirming that the molecular clock dating strategy was reliable. The divergence between *Lamioideae* and *Scutellarioideae* is about 29.45 million years ago (Mya), whereas the divergence between *Lamioideae* and *Nepetoideae* is about 40.71 Mya. The *Pedaliaceae* (*Sesamum indicum*) and *Lamiaceae* (*P. cablin*) shared a common ancestor during the Paleocene, around 50.03 Mya.

### 2.4. SSRs (Simple Sequence Repeats) Analysis

SSRs (simple sequence repeats), also called microsatellites, are tandem sequences that are widely distributed across the entire genome. SSRs are important signatures of a genome that has been widely used in genetic and genomic studies [22,23]. We detected perfect SSRs in chloroplast genomes of *P. Cablin*, as well as several other plants in *Lamiales* order. The number and types of chloroplast SSRs vary in different species (Table 3 and Table S8). *P. cablin* has the highest number of SSRs (52), whereas *Boea hygrometrica* has the lowest number of SSRs (11). The most prevalent SSRs among all the species are mono-nucleotide SSRs, which varied from six in *Boea hygrometrica* to 46 in *P. cablin*. Bi-nucleotide SSRs and compound SSRs are quite rare. We noticed that most of the mono-nucleotide SSRs are typically comprised of short polyA or polyT repeats, while tandem guanine (G) or cytosine (C) repeats are quite rare, which are in concordance with the results of other studies [16,24].

## 3. Materials and Methods

### 3.1. Plant Material and Library Preparation

Wild *P. cablin* plants were collected from Fenglai village, Yangchun City, Guangdong Province, China. Total chloroplast DNA was extracted from fresh leaves using Tiagen Plant Genomic DNA Kit (Beijing, China). Genomic DNA was fragmented into 300-bp using Covaris M220 Focused-ultrasonicator (Covaris, Woburn, MA, USA). Library preparation was conducted using NEBNext^®^ Ultra™ DNA Library Prep Kit Illumina (New England, Biolabs, Ipswich, MA, USA). Briefly, fragments were end-repaired (End Repair Reaction Buffer (10×) and End Prep Enzyme Mix), ligated with adaptors (NEBNext Adaptor and Blunt/TA Ligase Master Mix), removed uracil nucleotides from adapters, and purified (AMPure XP Beads, New England, Biolabs, Ipswich, MA, USA). The ligated DNA (with a 300-bp insertion) was then amplified by 6 cycles of PCR (Universal PCR Primer and Index (X) Primer; pre-denature 98 °C for 10 s, denature 98 °C 10 s, annealing 60 °C 30 s, elongation 72 °C 30 s). Finally, the PCR products were purified using AMPure XP Beads.

### 3.2. DNA Sequencing, Data Preprocessing and Genome Assembly

Cluster generation was performed using TruSeq PE Cluster Kit (Illumina, San Diego, CA, USA). Paired-end sequencing (2 × 125 bp) was carried out on an Illumina HiSeq 2500 platform. The raw reads contained adaptors and low-quality bases. FASTX-Toolkit [25] was used to remove adaptors, and trimmed 3′-low-quality bases that had quality scores <20 or ambiguous represented as “N”. A read and its paired read were removed when it was shorter than 20 bp after trimming. The remaining reads were paired by a home-made script. The final reads were called “clean reads”.

We used Velvet v1.2.07 [26] to perform *de novo* assembly of the chloroplast genome (Kmer is 101; other parameters: -ins_length 300, -scaffolding yes, -min_contig_lgth 500, -exp_cov 30). To complete the genome, gap closing was performed. The obtained contigs were mapped to the chloroplast genome of *Salvia miltiorrhiza*, a species also belonging to the Lamiaceae family, to determine the order and orientation of the contigs. We then carefully designed 9 pairs of primers that spanned boundaries of adjacent contigs to perform PCR amplification of the gap regions. PCR products were purified and then Sanger sequenced. The sequencing results and the contigs from the previous assembly were assembled using Lasergene SeqMan program from DNASTAR, Inc. (Madison, WI, USA). Finally, we obtained a high-quality complete *P. cablin* chloroplast genome, and the result was submitted to NCBI (Accession Number: KX230834).

### 3.3. Genome Annotation and Comparative Genomics

CpGAVAS [27] was used to predict genes and perform genome annotation, which was followed by manually checking for duplicate annotations. The predicted genes were also mapped to the nr database of the National Center for Biotechnology Information (NCBI), Kyoto Encyclopedia of Genes and Genomes (KEGG) [28], and the COG [29] database. We compared the chloroplast genome of *P. cablin* with those of *Origanum vulgare* L. and *Salvia miltiorrhiza*, two closely related species whose chloroplast genome have been completely sequenced, by MUMmer 3.0 [30]. The annotated chloroplast genomes of *Origanum vulgare* L. and *Salvia miltiorrhiza* were downloaded from NCBI. A gene map was drawn using GenomeVx [31]. To verify if any *trnK-UUU* genes were missing, we annotated the chloroplast genome by DOGMA [32].

### 3.4. Phylogenetic Tree Reconstruction and Divergence Time Estimation

The whole chloroplast genome of *P. cablin* was aligned to the complete chloroplast genomes of eight *Lamiaceae* plants and three outgroups from *Lamiales* (*Sesamum indicum*, *Utricularia gibba* and *Boea hygrometrica*) (Supplementary Table S9). Then, these nucleotide alignments were subjected to phylogenetic analyses with PhyML [33] using GTR + Ι + Γ substitution model. Estimation of divergence times was performed using the MCMCTree program in the PAML4.7 package under a relaxed clock model [34]. The “Independent rates model (clock = 2)” and “JC69” model in MCMCTree were used in our calculation. The MCMC procedures had a burn-in of 2,000,000 iterations and then run for 4,000,000 iterations. The default settings were adopted for other parameters when performing MCMCTree analysis. MCMCTree analysis was performed twice, which generated similar results confirming the robustness of the results. Chronogram was drawn using FigTree v1.4.0 (http://tree.bio.ed.ac.uk/) with the first run. We selected 120 and 130 Mya as the lower and upper boundaries for the Eurosid–Asterid split (*Arabidopsis*–tomato) [21].

### 3.5. SSR Identification

MIcroSAtellite identification tool [35] (MISA) was utilized to identify perfect SSRs in *P. cablin* chloroplast genome together with 11 other chloroplast genomes in order *Lamiales*. The settings included the following: more than 10 repeats for mono-nucleotide SSRs, six repeats for di-nucleotide SSRs, five repeats for tri-nucleotide SSRs, five repeats for tetra-nucleotide SSRs, five repeats for penta-nucleotide SSRs and five repeats for hexa-nucleotide SSRs. Compound SSRs were defined as two SSRs with <100 nt interspace nucleotides.

## 4. Conclusions

In this work, we presented the whole chloroplast genome of medicinal plant *P. cablin*. This genome is 152,460 bp in length, with similar quadripartite structure and genomic contents when compared with other species in the *Lamiaceae* family. *P. cablin* was diverged from the *Scutellarioideae* clade about 29.45 million years ago based on phylogenic analysis of chloroplasts. We also found that it has relatively more SSRs compared with other *Lamiaceae* members. Overall, the sequences and annotation of *P. cablin* chloroplast genome will facilitate phylogenic, population and genetic engineering research investigations of this species.

## Figures and Tables

**Table 1:** The introns and exons length of intron-containing genes.

Gene	Strand	Start	End	Exon I	Intron I	Exon II	Intron II	Exon III
*ndhB*	+	13,161	15,372	867	589	756		
*rpl2*	+	24,040	25,529	391	631	468		
*atpF*	−	37,673	38,945	150	664	459		
*rpoC1*	−	46,797	49,641	454	769	1622		
*ycf3*	−	67,542	69,472	134	696	235	720	146
*clpP*	−	95,175	97,129	74	726	294	638	223
*rpl2*	−	109,281	110,769	394	628	467		
*ndhB*	−	119,440	121,648	870	586	753		
*ndhA*	−	143,869	146,015	556	1055	536		

**Table 2:** Codon usage of *P. cablin* chloroplast genome.

AA	Codon	No.	Total	AA Frequency	AA	Codon	No.	Total	AA Frequency
A	GCA	399	1418	5.25%	P	CCA	318	1139	4.22%
	GCC	251				CCC	246		
	GCG	176				CCG	178		
	GCT	592				CCT	397		
C	TGC	88	319	1.18%	Q	CAA	702	925	3.42%
	TGT	231				CAG	223		
D	GAC	179	1024	3.79%	R	AGA	485	1639	6.07%
	GAT	845				AGG	197		
E	GAA	1002	1358	5.03%		CGA	367		
	GAG	356				CGC	121		
F	TTC	551	1539	5.70%		CGG	143		
	TTT	988				CGT	326		
G	GGA	736	1823	6.75%	S	AGC	131	2128	7.88%
	GGC	197				AGT	409		
	GGG	327				TCA	405		
	GGT	563				TCC	370		
H	CAC	146	640	2.37%		TCG	229		
	CAT	494				TCT	584		
I	ATA	703	2319	8.58%	T	ACA	413	1388	5.14%
	ATC	493				ACC	274		
	ATT	1123				ACG	164		
K	AAA	1046	1440	5.33%		ACT	537		
	AAG	394			V	GTA	557	1518	5.62%
L	CTA	420	2916	10.79%		GTC	178		
	CTC	203				GTG	223		
	CTG	213				GTT	560		
	CTT	616			W	TGG	497	497	1.84%
	TTA	881			Y	TAC	203	978	3.62%
	TTG	583				TAT	775		
M	ATG	619	619	2.29%	Stop codon	TAA	60	148	0.55%
N	AAC	310	1245	4.61%		TAG	42		
	AAT	935				TGA	46		

**Table 3:** Statistics of chloroplast SSRs (simple sequence repeats) detected in 12 species in *Lamiales*.

Species	Total	c	p2	p1						
				all	(A)10	(A)11	(A)12	(T)10	(T)11	(T)12
*Pogostemon cablin*	52	6	0	46	11	6	3	10	6	2
*Scutellaria baicalensis*	26	3	2	21	2	3	0	5	5	1
*Scutellaria insignis*	26	1	2	23	4	1	0	5	2	0
*Premna microphylla*	39	4	1	34	8	4	1	8	3	5
*Tectona grandis*	33	2	2	29	9	1	0	10	1	1
*Lavandula angustifolia*	27	4	3	20	3	2	0	7	3	0
*Salvia miltiorrhiza*	30	2	1	27	3	3	1	5	3	4
*Rosmarinus officinalis*	20	2	2	16	4	1	0	6	3	1
*Origanum vulgare* L.	29	2	1	26	3	1	3	4	6	2
*Sesamum indicum*	23	0	2	21	6	0	0	7	5	0
*Utricularia gibba*	28	1	1	26	9	4	0	8	3	0
*Boea hygrometrica*	11	0	2	9	4	0	0	3	0	0

c, compound SSRs; p2, di-nucleotide SSRs; p1, mono-nucleotide SSRs. A, Adenine; T, Thymine, G, Guanine; C, Cytosine.

## Supplementary Information

Supplementary materials can be found at http://www.mdpi.com/1422-0067/17/6/820/s1.
