# Supplementary material for: The Complete Chloroplast Genome Sequences of the Medicinal Plant Pogostemon cablin
Source: Int J Mol Sci. 2016 Jun 6;17(6):820. doi: 10.3390/ijms17060820 (PMC4926354; doi:10.3390/ijms17060820)
Supplement: Supplementary file 1 [file ijms-17-00820-s001.zip › ijms-127609-Supplementary Figure S1 and Table S9 (update).pdf]

# Supplementary Materials: The Complete Chloroplast Genome Sequences of the Medicinal Plant *Pogostemon cablin*

**Yang He, Hongtao Xiao, Cao Deng, Liang Xiong, Jian Yang and Cheng Peng**

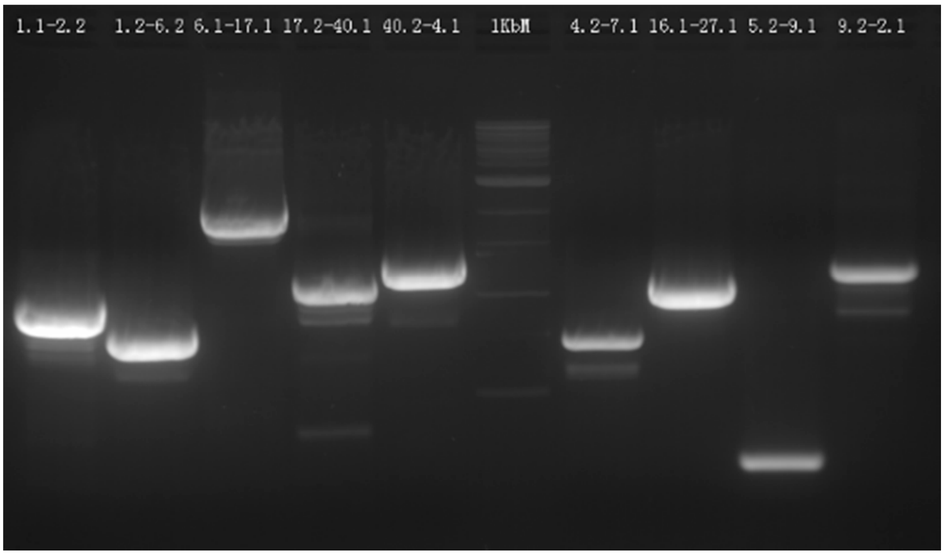


**Figure S1.** PCR products on agarose gel electrophoresis. Each lane represents the PCR product of gap area (Table S2), except that the "1KbM" is the marker lane.

**Table S9.** Species used to reconstruct phylogenetic tree and estimate divergence time.

| Species | Accession Number | Note |
|---|---|---|
| *Premna microphylla* | NC_026291.1 | |
| *Scutellaria insignis* | NC_028533.1 | |
| *Lavandula angustifolia* | NC_029370.1 | |
| *Salvia miltiorrhiza* | NC_020431.1 | |
| *Scutellaria baicalensis* | NC_027262.1 | |
| *Origanum vulgare* subsp. *vulgare* | JX880022.1 | |
| *Rosmarinus officinalis* | NC_027259.1 | |
| *Tectona grandis* | NC_020098.1 | |
| *Sesamum indicum* | NC_016433.2 | Out group |
| *Utricularia gibba* | NC_021449.1 | Out group |
| *Boea hygrometrica* | NC_016468.1 | Out group |
